# U2AF65-Dependent SF3B1 Function in SMN Alternative Splicing

**DOI:** 10.3390/cells9122647

**Published:** 2020-12-09

**Authors:** Namjeong Choi, Yongchao Liu, Jagyeong Oh, Jiyeon Ha, Xuexiu Zheng, Haihong Shen

**Affiliations:** School of Life Sciences, Gwangju Institute of Science and Technology, Gwangju 500-712, Korea; njchoi@gist.ac.kr (N.C.); yongchao@gist.ac.kr (Y.L.); jgoh@gist.ac.kr (J.O.); hajiyn@gist.ac.kr (J.H.)

**Keywords:** SF3B1, alternative splicing, SMN, spinal muscular atrophy

## Abstract

Splicing factor 3b subunit 1 (SF3B1) is an essential protein in spliceosomes and mutated frequently in many cancers. While roles of SF3B1 in single intron splicing and roles of its cancer-linked mutant in aberrant splicing have been identified to some extent, regulatory functions of wild-type SF3B1 in alternative splicing (AS) are not well-understood yet. Here, we applied RNA sequencing (RNA-seq) to analyze genome-wide AS in SF3B1 knockdown (KD) cells and to identify a large number of skipped exons (SEs), with a considerable number of alternative 5′ splice-site selection, alternative 3′ splice-site selection, mutually exclusive exons (MXE), and retention of introns (RI). Among altered SEs by SF3B1 KD, survival motor neuron 2 *(SMN2)* pre-mRNA exon 7 splicing was a regulatory target of SF3B1. RT-PCR analysis of *SMN* exon 7 splicing in SF3B1 KD or overexpressed HCT116, SH-SY5Y, HEK293T, and spinal muscular atrophy (SMA) patient cells validated the results. A deletion mutation demonstrated that the U2 snRNP auxiliary factor 65 kDa (U2AF65) interaction domain of SF3B1 was required for its function in *SMN* exon 7 splicing. In addition, mutations to lower the score of the polypyrimidine tract (PPT) of exon 7, resulting in lower affinity for U2AF65, were not able to support SF3B1 function, suggesting the importance of U2AF65 in SF3B1 function. Furthermore, the PPT of exon 7 with higher affinity to U2AF65 than exon 8 showed significantly stronger interactions with SF3B1. Collectively, our results revealed SF3B1 function in *SMN* alternative splicing.

## 1. Introduction

Introns are removed from pre-mRNA during splicing by the spliceosome, a protein–RNA complex that catalyzes the excision of introns and the ligation of exons to form mature mRNA [1,2]. The spliceosome assembles onto pre-mRNA with five small nuclear ribonucleoproteins (snRNPs) (U1, U2, U4, U5, and U6) and non-snRNPs [1]. Pre-mRNA splicing involves two consecutive transesterification steps: in the first step, the adenosine from the branchpoint site (BPS) attacks the 5′ splice site (5′SS) of the intron to cleave 5′SS and form an intron lariat; in the second step, the 3’ hydroxyl group attacks the 3′ splice site (3′SS) to cleave 3′SS with concurrent ligation of two exons [1]. In the early stage of spliceosome assembly, the BPS, 3′SS, and the middle polypyrimidine tract (PPT) are bound by proteins or RNA–protein complexes cooperatively. U2 snRNP auxiliary factor 35 kDa (U2AF35) binds to the 3′SS, U2 snRNP auxiliary factor 65 kDa (U2AF65) binds to the PPT with extensive interaction with BPS, and splicing factor 1 (SF1) recognizes BPS [3,4,5]. Cooperative binding of these proteins to pre-mRNA facilitate the recruitment of U2 snRNP to the 3′SS [2]. Base-pairing between U2 snRNA and BPS is weak; thus, supportive stabilization by additional factors is necessary. The first support is from splicing factor 3b (SF3B), a multiprotein component of the U2 snRNP that can interact with pre-mRNA at or near the BPS to reinforce the base-pairing between U2 snRNP and BPS [6,7]. The second support is from the interaction between the arginine/serine-rich (RS) domain of U2AF65 and the BPS to strengthen the base-pairing of BPS and U2 snRNA [4].

SF3B1 (or SF3B155) is the largest (155 kDa) core component of SF3B. The N-terminal of SF3B1 comprises multiple U2AF65 binding motifs called ULMs (U2AF homology motif (UHM) ligand motifs) that can interact with UHM to facilitate the recruitment of U2 snRNP to the BPS [8,9]. Two-thirds of the C-terminal of SF3B1 includes 20 tandem HEAT (huntingtin, elongation factor 3, subunit A of protein phosphatase 2A, and phosphatidylinositol 3-kinase (PI3K) target of rapamycin 1) repeats that serve as scaffolds for interactions with other SF3B subunits by forming helical structures [10,11]. SF3B1 with p14, another U2 snRNP component, can directly interact with BPS adenosine [3,12]. It has been shown that SF3B1 can directly contact pre-mRNA on both sides of the BPS [7,13]. These interactions of SF3B1 with U2AF65, p14, and pre-mRNA suggest that interactions among SF3B1, U2AF65, and p14 are essential for the recognition of BPS by the spliceosome. 

Alternative splicing (AS) increases genome diversity by producing multiple mRNA isoforms from a single gene [14]. AS is dysregulated in multiple diseases, including cancer and genetic diseases [14,15,16,17,18,19]. Aberrant splicing in cancer can be caused by misrecognition of BPS by U2 snRNA [20,21]. SF3B1 is the most frequently mutated component of a spliceosome associated with diseases such as chronic lymphocytic leukemia (CLL), chronic myelomonocytic leukemia (CMML), myelodysplastic syndrome (MDS), and breast and pancreatic cancers [22,23,24,25,26,27,28] and is the best-known drug target in spliceosomes [28]. Recurrent hotspot mutations of SF3B1 are found exclusively in the C-terminal HEAT-repeat domain (HD), and cause alternative usage of BPS and cryptic 3′SS [21,29,30]. A recent study has shown that a disease-causing SF3B1 mutant can hinder its interaction with SURP and G patch domain containing 1 (SUGP1) to cause aberrant splicing [31]. Spliceostatin A that targets SF3B1 can severely affect the fidelity of BPS and 3′SS selection [32].

Spinal muscular atrophy (SMA), the second most prevalent genetic cause of infant mortality, is an autosomal recessive genetic disease, in which motor neurons in the anterior horn of the spinal cord are severely damaged [33]. SMA is caused by a deletion or mutation in survival motor neuron 1 (*SMN1*) gene, a telomeric copy in chromosome 5, coding for survival of motor neuron (SMN) protein [34]. SMN has a canonical role in the assembly of Sm proteins onto snRNAs to form U snRNP protein–RNA complexes [35,36,37]. The ability of SMN in assembling Sm proteins onto snRNAs is highly correlated with SMA severity in cells [38]. Low amounts of SMN in axon lead to reduced β-actin mRNA transport and axon defects [38]. A duplicate of *SMN1* gene called *SMN2* is found in all of the SMA patients. However, it mostly produces an mRNA with cassette exon 7 exclusion (~80–90%) that encodes a truncated protein and highly unstable isoform (SMNΔ7) that does not support SMN function [39,40]. C6U transition in *SMN2* exon 7 compared to *SMN1* gene has been demonstrated to cause exon 7 skipping [39,40]. Various mechanisms, including abrogation of a positive element, creation of a negative element, and formation of an inhibitory context, have been attributed to C6U transition within *SMN2* exon 7 [41]. Therapies for SMA patients include increasing the production of SMN through modifying *SMN2* splicing or gene therapy by delivering *SMN1* gene [42,43,44,45,46]. Nusinersen is the first FDA-approved drug for SMA, which is an antisense oligonucleotide-based compound targeting the intronic splicing silencer and thereby regulating the splicing of *SMN2* exon 7 [46]. However, nusinersen is not able to cross the blood–brain barrier; thus, repeated intrathecal injection is necessary for therapy [46]. Compared with nusinersen, AVXS-101, an adeno-associated viral vector that forms a double-stranded DNA template to deliver *SMN1* to target motor neurons, can cross the blood–brain barrier [46]. However, none of the available approaches have the potential to fully cure SMA [47]. Thus, a deeper understanding of the molecular mechanism of *SMN1/2* splicing is necessary.

Studies on SF3B1 conducted thus far have revealed the role of multiple domains in defining the appropriate BPS close to the 3′SS [48]. However, regulatory functions of wild-type SF3B1 in AS are not well-understood yet. Here, using RNA sequencing (RNA-seq), genome-wide AS in SF3B1 knockdown (KD) cells was analyzed. A large number of skipped exons (SEs) and a considerable number of alternative 5′ splice-site selection (A5SS), alternative 3′ splice-site selection (A3SS), mutually exclusive exons (MXE), and retention of introns (RI) were identified. Among altered SEs by SF3B1 KD, *SMN2* pre-mRNA exon 7 splicing was identified as a regulatory target of SF3B1. An RT-PCR analysis of *SMN* exon 7 splicing in SF3B1 KD or overexpressed HCT116, SH-SY5Y, HEK293T, and SMA patient cells validated the results. A deletion mutation demonstrated that the U2AF65 interaction domain of SF3B1 was required for its function in *SMN* exon 7 splicing. In addition, mutations to lower the score of the PPT of exon 7, resulting in lower affinity for U2AF65, were unable to support SF3B1 function, further suggesting the importance of U2AF65 in SF3B1 function. Furthermore, the PPT of exon 7 with higher affinity for U2AF65 than exon 8 showed significantly stronger interactions with SF3B1. Collectively, our results revealed the important function of SF3B1 in *SMN* alternative splicing.

## 2. Materials and Methods

### 2.1. Cell Culture, Transfection, and shRNA Virus Treatment

HCT116 cells were grown in Roswell Park Memorial Institute Medium (RPMI) supplemented with 10% fetal bovine serum (FBS), 2 mM glutamine, 100 U/mL penicillin, and 100 μg/mL streptomycin at 37 °C in a 5% CO_2_ incubator. SH-SY5Y, HEK293T, and SMA type I fibroblast GM03813 (Coriell Repositories, Camden, NJ, USA) cell lines were grown in Dulbecco’s Modified Eagle’s Medium (DMEM) as previously described [18]. Plasmids were transfected into cells using the polyethylenimine (PEI) (Sigma, St. Louis, MO, USA) reagent as previously described [18]. Total RNA was extracted at 48 h post-transfection for RT-PCR. An shRNA virus was produced in HEK293T cells by transfecting an shRNA plasmid (Open Biosystems, Huntsville, AL, USA) DNA along with psPAX2 (the packaging vector) and pMD2.G (the envelope vector) with PEI treatment as previously described [18]. The supernatant containing viral particles was filtered through a 0.45 μm filter. HCT116, SH-SY5Y, HEK293T, and GM03813 cells were infected by the shRNA virus with 5 mg/mL polybrene (Sigma, St. Louis, MO, USA) treatment as previously described [18]. Total RNA was extracted after 72 h of infection for subsequent RT-PCR analysis.

### 2.2. RNA Extraction and RT-PCR

Total RNA was extracted from cells using the RiboEX reagent (GeneAll, Lisbon, Portugal) following the manufacturer’s instructions as previously described [18]. Total RNA (1 μg) was then reverse transcribed to cDNA using moloney murine leukemia virus (M-MLV) reverse transcriptase (Elpis) with oligo-dT18 primer as previously described [49]. PCR was then performed with cDNA (1 µL) using gene-specific primers. PCR products were loaded onto 2% agarose gels and visualized using ethidium bromide (EtBr) staining. Quantitative RT-PCR (RT-qPCR) was performed using the KAPA SYBR FAST kit (KK4606) according to manufacturer’s instructions with β-actin (ACTB) as an internal control. A multi-exon skipping detection assay (MESDA) was performed with unlabeled primers annealing to exon 2b and exon 8 as previously described [50]. Primers used in PCR reactions are listed in Supplementary Table S1.

### 2.3. RNA-Sequencing (Seq) and Bioinformatical Analysis

Purification of mRNAs and construction of the cDNA library with total RNA from non-silencing or SF3B1 shRNA-treated HCT116 cells were performed by Macrogen Inc. (Korea). High-throughput paired-end 100-nucleotide (nt) sequencing was performed using the Illumina NovaSeq platform (Macrogen, Seoul, Korea). The replicate multivariate analysis of transcript splicing (rMATS) software was applied to compare AS of SF3B1 KD with the control [51]. The rMATS output was filtered with the following criteria: *p* < 0.05 and Δpercent-splice-in (ΔPSI) > 10%. Gene ontology (GO) analysis for regulation of AS by SF3B1 KD was performed using DAVID Bioinformatics Resources 6.8 (https://david.ncifcrf.gov/) [52]. Primers used for validations of RNA-seq results are shown in Supplementary Table S1.

### 2.4. Construction of Plasmids

pCDNA3.1-flag-SF3B1-WT was a gift from Manoj Pillai (Addgene plasmid # 82576; http://n2t.net/addgene:82576) [30]. The ΔULM mutant plasmid was constructed using overlapping PCR and inserted into the BamHI/EcoRI restriction enzyme site of the pCDNA3.1-flag plasmid.

### 2.5. Immunoblotting, RNA Pull-Down/Immunoprecipitation, and UV Crosslinking/Immunoblotting Assay

Cells were lysed with lysis buffer (0.1% Triton X-100, 50 mM Tris-Cl (pH 7.5), 150 mM NaCl, 5 mM EDTA, 1 mM β-mercaptoethanol) for 30 min at 4 °C as previously described [18]. Cell lysates were centrifuged at 12,000 rpm for 15 min at 4 °C. Supernatants were obtained, loaded onto SDS-PAGE for separation, and then transferred to polyvinylidene fluoride (PVDF) membranes. Anti-SF3B1 (Abcam, ab172634) and anti-tubulin (Abcam, ab18251) antibodies were used for immunoblotting analysis. RNA pull-down/immunoblotting analysis was performed as previously described [18]. Briefly, biotin-labeled oligonucleotides were covalently linked to streptavidin agarose (Millipore) in buffer D (20 mM Tris-Cl (pH7.5), 150 mM KCl, 0.2 mM ethylenediamenetetraacetic acid (EDTA), 10% glycerol, 0.5 mM dithiothreitol (DTT), 0.5 mM phenylmethylsulfonyl fluoride (PMSF)) for 1 h at 4 °C. Cell lysates were mixed with RNA-linked beads in buffer D for 4 h at 4 °C, washed with buffer D for five times, loaded onto 6% SDS-PAGE gel, and then subjected to immunoblotting. A UV crosslinking/immunoblotting analysis was performed as described previously [18]. In brief, UV light at 80,000 µJ from Stratalinker (Stratagene) was applied for 5 min to crosslink biotin-labeled RNA and proteins in cell lysates followed by immunoprecipitation with anti-SF3B1 (Bethyl, A300-996A) and immunoblotting with horseradish peroxidase (HRP)-conjugated streptavidin (RABHRP3).

### 2.6. Statistical Analysis

RT-PCR, immunoblotting, and immunoprecipitation analyses were performed in triplicate. Data are presented as mean ± SD (standard deviation of the mean) and the statistical differences among groups were analyzed using the one-way ANOVA tool. Statistical significance was shown as * *p* < 0.05, ** *p* < 0.01, *** *p* < 0.001, and **** *p* < 0.0001.

## 3. Results

### 3.1. RNA-Seq Reveals Global Effects of SF3B1 on AS

**Figure 1:**
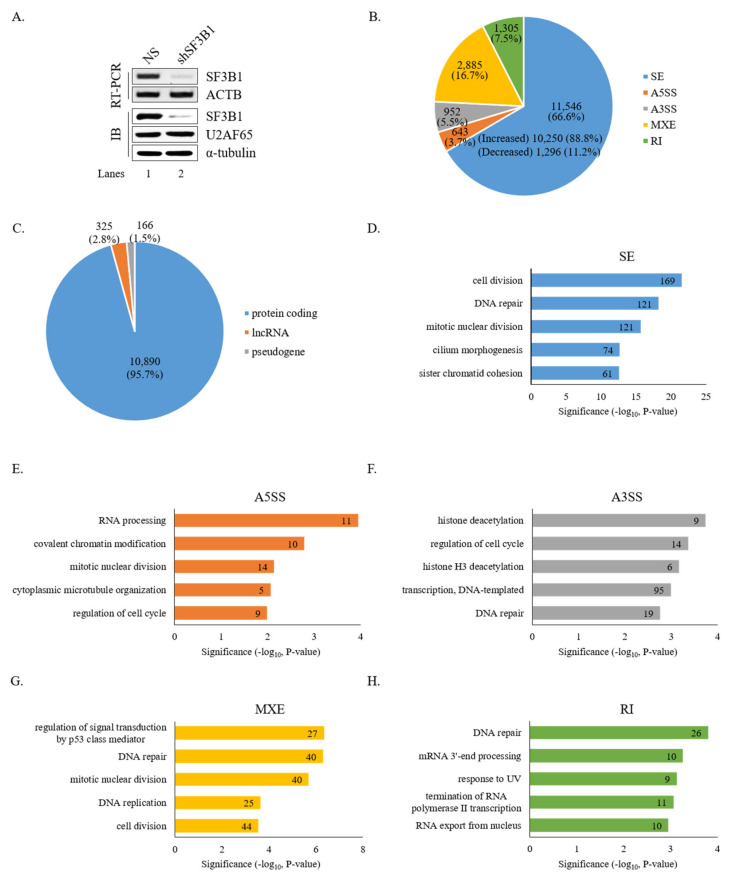
Global effects of SF3B1 on AS based on RNA-seq. (**A**) Reduced expression of SF3B1 in HCT116 cells treated with an SF3B1-targeting shRNA or a non-silencing shRNA based on RT-PCR and immunoblotting using ACTB for RT-PCR and α-tubulin and U2AF65 for immunoblotting controls. (**B**) Pie chart of RNA-seq results showing alteration quantities of SEs, A5SS, A3SS, MXE, and RI in SF3B1 KD cells. (**C**) Pie chart of gene distributions of altered SEs in SF3B1 KD cells. (**D**–**H**) Gene ontology analysis of AS-regulated genes in SEs, A5SS, A3SS, MXE, and RI of SF3B1 KD cells.

To gain insight into the roles of SF3B1 in AS, RNA-seq was performed using RNA from HCT116 cells treated with an SF3B1-targeting shRNA. As shown in Figure 1A, SF3B1 RNA and protein levels were significantly decreased by treatment with the SF3B1-targeting shRNA than in non-silencing shRNA-treated cells based on RT-PCR and immunoblotting analyses (lane 2). In addition, SF3B1-interacting U2AF65 protein expression level was not affected by SF3B1 KD (lane 2). Bioinformatical analysis using rMATS of RNA-seq results demonstrated that the AS of 11,546 SE events (10,250 increased and 1,296 decreased) was affected significantly (ΔPSI ≥ 10%) by SF3B1 KD (Figure 1B) (Supplementary Table S2). In addition to SE events, significant alterations of A5SS (*n* = 643), A3SS (*n* = 952), MXE (*n* = 2,885), and RI (*n* = 1,305) were also observed (Figure 1B) (Supplementary Table S2). Gene identity analysis showed that most of these genes with AS events affected by SF3B1 were protein-coding genes (~95.7%), although much smaller portions of long non-coding RNAs (lncRNAs) (~2.8%) and pseudogenes (~1.5%) were also affected (Figure 1C). GO analysis demonstrated that functions of cell division, DNA repair, and mitotic nuclear division were enriched for genes in SE category (Figure 1D); and RNA processing, covalent chromatin modification, and mitotic nuclear division were enriched in the A5SS category (Figure 1E). Histone deacetylation, regulation of cell cycle, and histone H3 deacetylation were enriched in the A3SS category (Figure 1F). Regulation of signal transduction by p53 class mediator, DNA repair, and mitotic nuclear division were enriched in the MXE category (Figure 1G). DNA repair, mRNA 3′-end processing, and response to UV were in the RI category (Figure 1H). Thus, the results above indicated that SF3B1 has widespread roles in AS.

**Figure 2:**
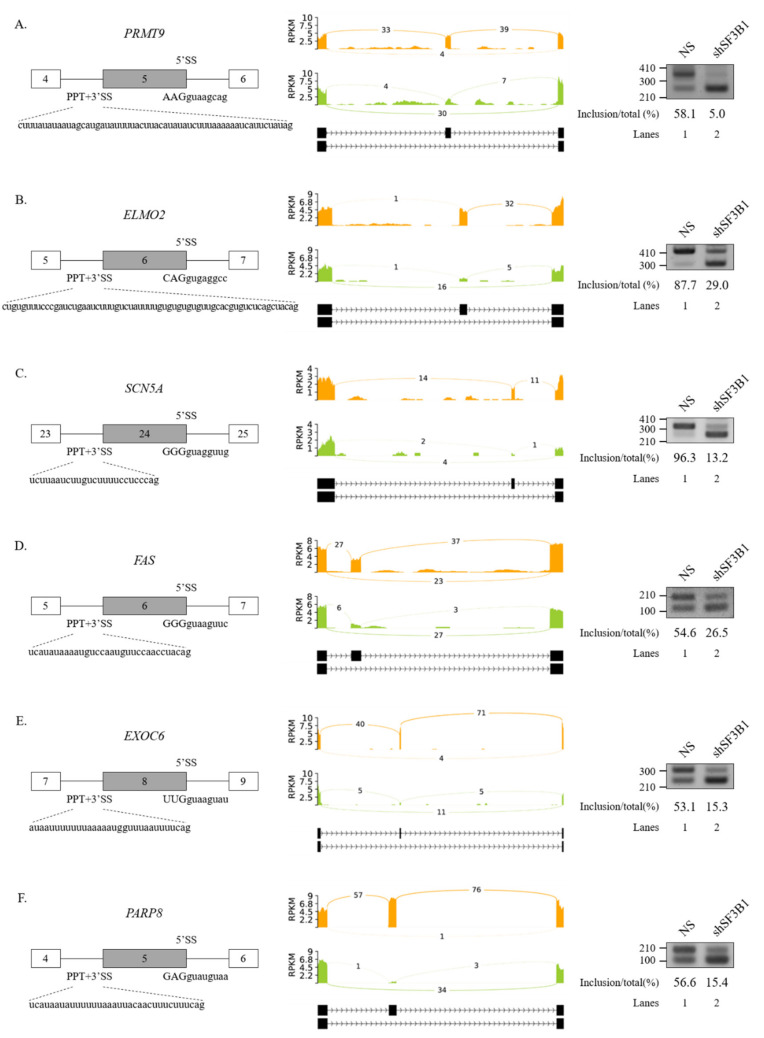
Validation of regulated SEs in SF3B1 KD cells. (**A**–**F**) (Left) Schematic representation of alternative exons in genes. Sequences of 5′SS, 3′SS, and PPT of cassette exons are shown. (Middle) Sashimi plots of validated SEs in RNA-seq. (Right) RT-PCR analysis of altered SEs in SF3B KD cells vs. control cells.

**Figure 3:**
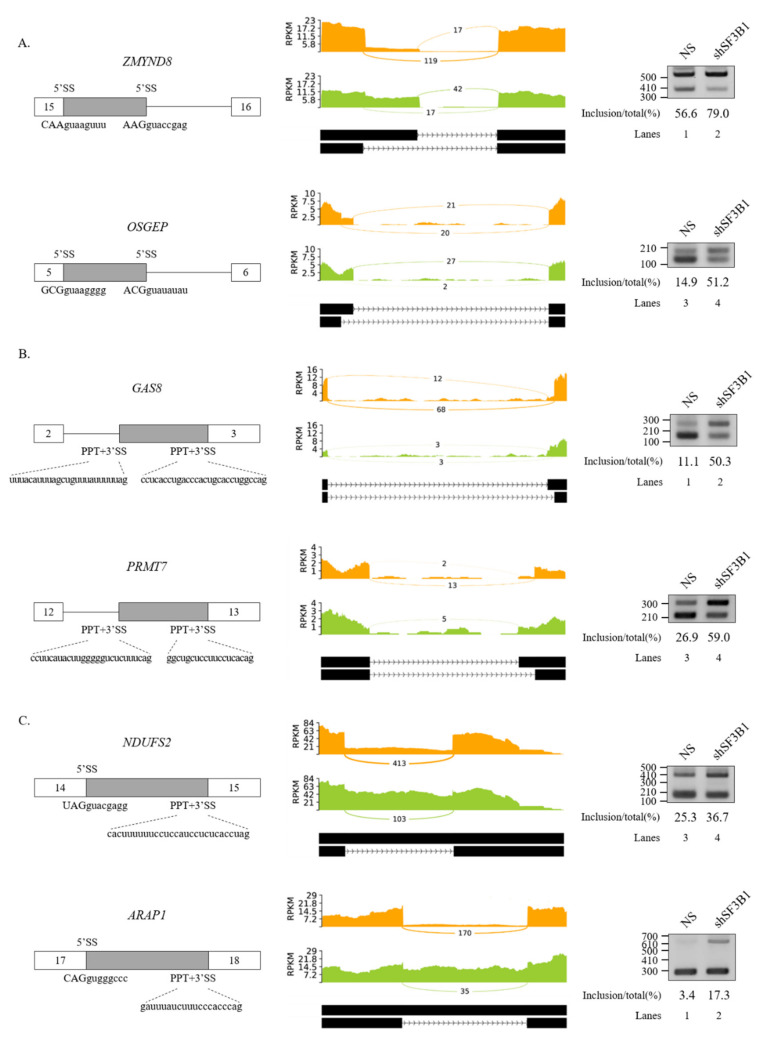
Validation of regulated A5SS, A3SS, and RI in SF3B1 KD cells. (**A**–**C**) (Left) Schematic representation of alternative splice sites in genes. Sequences of two alternative 5′SSs (**A**); two alternative 3′SSs and PPTs (**B**); and 5′SSs, 3′SSs, and PPTs of introns (**C**) are shown. (Middle) Sashimi plots of validated A5SS (**A**), A3SS (**B**), and RI (**C**) in RNA-seq. (Right) RT-PCR analysis of altered A5SS (**A**), A3SS (**B**), and RI (**C**).

We further performed RT-PCR analysis for 20 AS events that showed high ΔPSI value in various AS categories. Among them, 19 AS events showed significant alterations in the ratio of AS in total mRNA. As shown in Figure 2, RT-PCR results validated the following six SE events: protein arginine methyltransferase (*PRMT9*) (Figure 2A), engulfment and cell motility 2 (*ELMO2*) (Figure 2B), sodium voltage-gated channel alpha subunit 5 (*SCN5A*) (Figure 2C), Fas cell surface death receptor (*FAS*) (Figure 2D), exocyst complex component 6 (*EXOC6*) (Figure 2E), and poly(ADP-ribose) polymerase family member 8 (*PARP8*) (Figure 2F) genes. A5SS events (zinc finger MYND-type containing 8 (*ZMYND8*) and O-sialoglycoprotein endopeptidase (*OSGEP*)) (Figure 3A), A3SS events (growth arrest specific 8 (*GAS8*) and protein arginine methyltransferase 7 (*PRMT7*) genes) (Figure 3B), and RI events (ArfGAP with RhoGAP domain, ankyrin repeat, and PH domain 1 (*ARAP1*) and NADH:ubiquinone oxidoreductase core subunit S2 (*NDUFS2*) genes) (Figure 3C) were also validated. Collectively, these results revealed that SF3B1 can regulate various types of AS at extensive levels.

### 3.2. SF3B1 Regulates Cassette Exon Splicing of SMN1 and SMN2 Pre-mRNA

**Figure 4:**
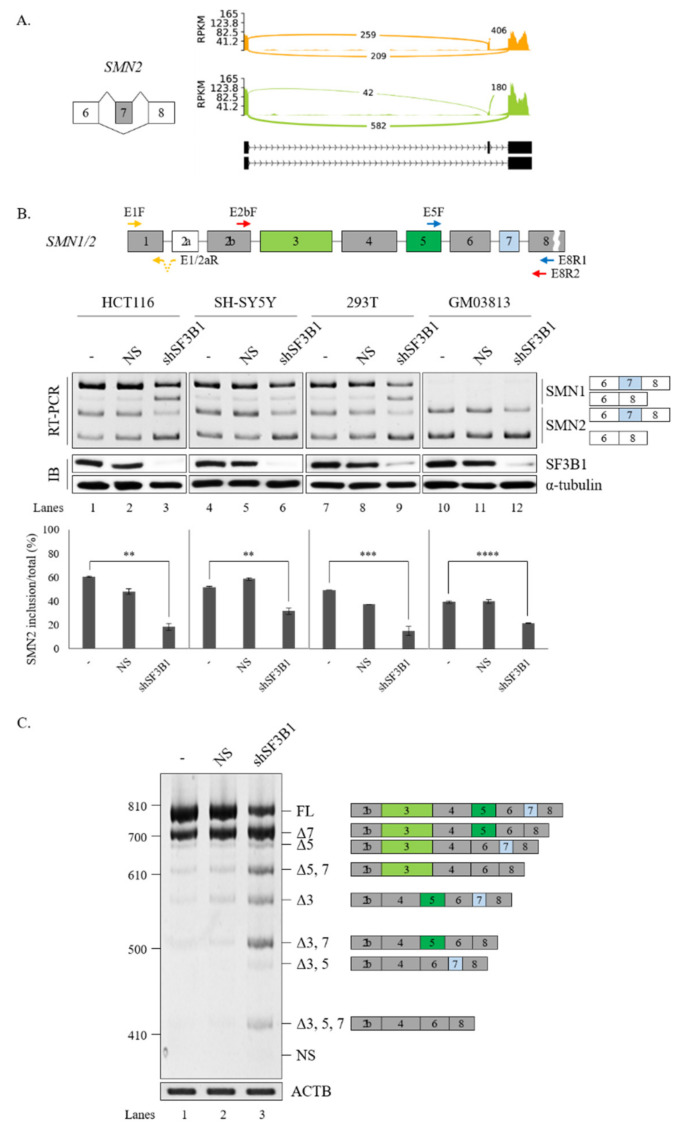
SF3B1 regulates AS of *SMN* pre-mRNA. (**A**) Sashimi plots of *SMN2* pre-mRNA splicing in RNA-seq of SF3B1 KD cells. (**B**) (Upper) location of primer annealing in RT-PCR. (Lower) RT-PCR analysis of *SMN1* and *SMN2* pre-mRNA splicing with primers E5F and E8R1 in HCT116, SH-SY5Y, HEK293T, and SMA patient (GM03813) cells treated with an SF3B1-targeting shRNA. Reduced SF3B1 expression levels by the shRNA are shown with RT-PCR and immunoblotting using ACTB and α-tubulin as controls. Statistical analysis results of RT-PCR are shown with *p*-values: **** *p* < 0.0001; *** *p* < 0.001; ** *p* < 0.01; and * *p* < 0.05. (**C**) Analysis of multiple exon splicing in HEK293T cells using the MESDA and primers E2bF and E8R2 with ACTB as a control. Alternatively spliced exons are labeled with green or blue colors.

A previous study showed the effect of pladienolide B, an inhibitor of SF3B1, on the splicing of *SMN2* exon 7 [53]. Among AS events regulated by SF3B1 in RNA-seq, we noticed that the reads of cassette exon 7 were reduced significantly and the reads of flanking exons were increased in SF3B1 KD (Figure 4A). To validate this RNA-seq result, RT-PCR analysis was performed for *SMN1* and *SMN2* mRNA in HCT116 cells treated with the SF3B1-targeting shRNA or the non-silencing shRNA (control). RT-PCR products of *SMN1* and *SMN2* were separated after cleavage with DdeI enzyme as previously described [18]. Consistent with RNA-seq results, cassette exon inclusion was significantly decreased in both *SMN1* and *SMN2* pre-mRNAs after SF3B1 KD (~69.4% and~18.4%, respectively) (Figure 4B, lane 3). Accordingly, cassette exon skipping was increased in both *SMN1* and *SMN2* pre-mRNAs. Next, it was determined whether SF3B1 KD effects could also be observed in other cells. As shown in Figure 3B, SF3B1 KD caused substantial decrease of exon 7 inclusion in SH-SY5Y cells derived from neuroblastoma patients (~31.4%) (lane 6) and HEK293T cells (~14.6%) (lane 9). SF3B1 KD also inhibited cassette exon inclusion in GM03813 fibroblast cells, derived from SMA patients, in which *SMN1* gene was deleted (~21.6%) (lane 12). Thus, reduced SF3B1 expression could inhibit cassette exon inclusion of *SMN1* and *SMN2* in various cell lines. As pladienolide B treatment also induced a decrease in the mRNAs of both exon 7-included and -skipped isoforms [53], we wondered whether SF3B1 KD induced transcript level alterations of *SMN*. To this aim, we performed RT-qPCR using primers to exon 1 and exon 1/2A boundary. As shown in Supplementary Figure S1, SF3B1 KD induced reduction of *SMN* transcript in HCT116 and SH-SY5Y cells (but not in HEK293T and SMA patient cells), suggesting that SF3B1 KD might inhibit *SMN* transcription or promote mRNA decay in specific cell lines.

We next applied MESDA [50] with primers annealing to exon 2b and exon 8 to determine if splicing of other *SMN* exons were also affected by SF3B1 KD. As shown in Figure 4C, skipping of exon 5 (Δ5), co-skipping of exons 5 and 7 (Δ5, 7), skipping of exon 3 (Δ3), and co-skipping of exons 3 and 7 (Δ3, 7) were significantly increased upon SF3B1 KD in HEK293T cells, indicating that, in addition to exon 7, SF3B1 KD also affected the splicing of various exons in *SMN* pre-mRNA.

**Figure 5:**
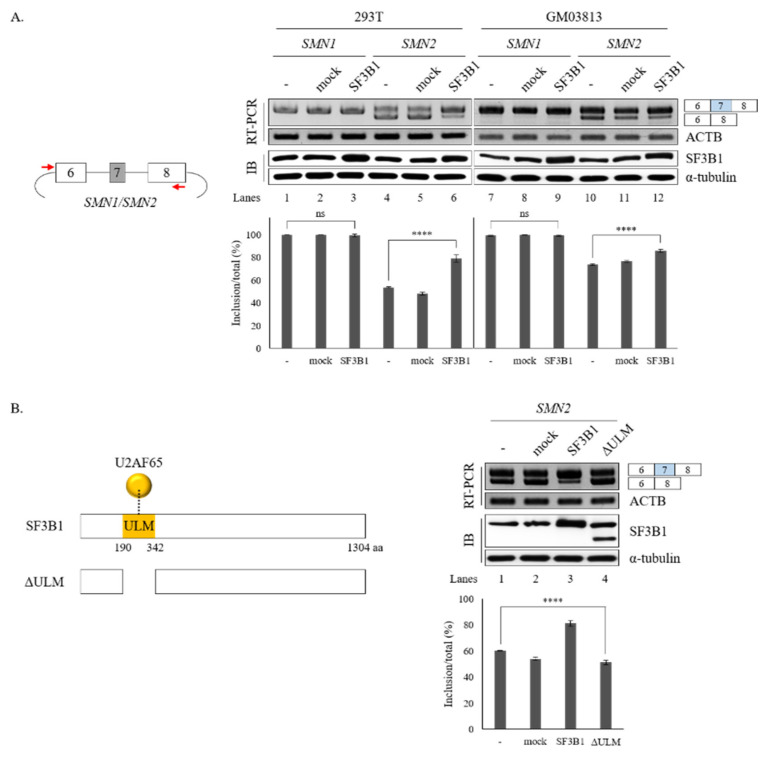
ULM is required for SF3B1 function in *SMN* exon 7 splicing. (**A**) (Left) Schematic representation of *SMN1* or *SMN2* minigenes. (Right) RT-PCR analysis of *SMN1* or *SMN2* pre-mRNA within minigenes in SF3B1-overexpressed cells or control cells transfected with empty plasmids using ACTB as a control. Expression levels of SF3B1 protein are shown with immunoblotting with α-tubulin as a control. Statistical analysis results of RT-PCR are shown with *p*-values: **** *p* < 0.0001; *** *p* < 0.001; ** *p* < 0.01; and * *p* < 0.05. (**B**) (Left) Schematic representation of ΔULM mutant SF3B1 protein. (Right) RT-PCR analysis of *SMN2* pre-mRNA in cells overexpressed with wild-type and ΔULM mutant SF3B1. Quantitation results are shown.

We further wondered whether increased SF3B1 expression might have effects opposite to the effects of SF3B1 KD on the splicing of *SMN1* or *SMN2*. To address the question, *SMN1* and *SMN2* minigenes produced in our group previously [18] were applied. As SF3B1 KD inhibited cassette exon skipping in both *SMN1* and *SMN2* pre-mRNA, we expected that SF3B1 overexpression could stimulate exon 7 inclusion. As reported previously [18], the *SMN1* minigene exclusively produced an exon 7-included isoform. Therefore, further increasing of exon 7 inclusion would be impossible. As shown in Figure 5A, overexpression of SF3B1 did not change AS in the *SMN1* minigene in either HEK293T or GM03813 cells, independently, as expected (lanes 3 and 9). In contrary to the *SMN1* minigene, the *SMN2* minigene produced an exon 7-skipped isoform mostly (lane 4). SF3B1 overexpression significantly stimulated exon 7 inclusion in both HEK293T and GM03813 cells (~79.0% and ~85.8%, respectively) (lanes 6 and 12), in contrast to SF3B1 KD effects (Figure 5A). Therefore, SF3B1 overexpression can promote cassette exon inclusion of *SMN2* pre-mRNA. Taken together, these results indicated that SF3B1 is a regulatory factor of *SMN* pre-mRNA splicing.

### 3.3. Interaction of SF3B1 with U2AF65 is Required for SF3B1 Function in SMN Exon 7 Splicing

It has been shown that SF3B1 can interact with U2AF65 through its N-terminal ULM domain (190–342 amino acids (aa)) to enable the recruitment of U2 snRNP to the BPS [8,9] (Figure 5B, left). Thus, we wondered whether the ULM domain of SF3B1 might be required for its regulation of *SMN* exon 7 splicing.

To address this question, we produced a ΔULM mutant of SF3B1, in which ULM domains were deleted (Figure 5B, left). This mutant was then overexpressed in cells harboring the *SMN2* minigene. As shown in Figure 5B (right), the ΔULM mutant could not promote cassette exon inclusion (lane 4) as wild-type SF3B1 (lane 3), although similar amounts of ΔULM proteins were expressed from these mutants as the wild-type SF3B1 expression vector (lanes 3 and 4). This indicated that the ULM domain is required for SF3B1 function in *SMN2* splicing. Therefore, we can conclude that the interaction of SF3B1 with U2AF65 is required for the regulating role of SF3B1 in the splicing of *SMN2* pre-mRNA.

### 3.4. PPT Sequences of Cassette Exon Are Essential for SF3B1 Function in SMN Exon 7 Splicing

**Figure 6:**
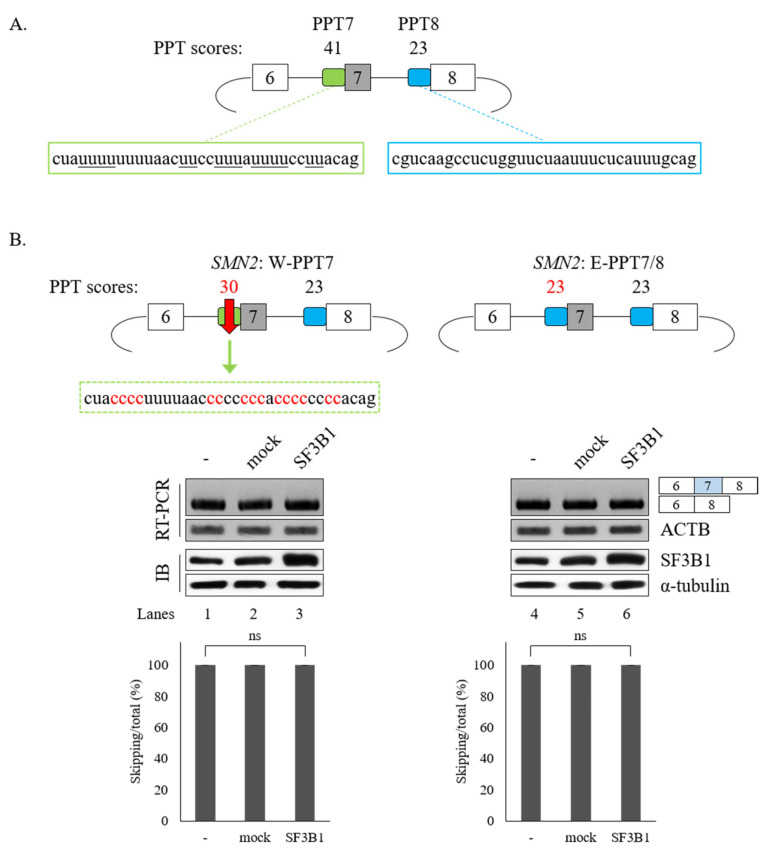
PPT sequences of cassette exon are essential for SF3B1 function in *SMN* exon 7 splicing. (**A**) Schematic representation of *SMN2* minigene with PPT scores and sequences in exon 7 and exon 8. (**B**) (Upper) PPT scores and sequences of W-PPT and E-PPT7/8 mutants of *SMN2* pre-mRNA. (Lower) RT-PCR analysis of W-PPT and E-PPT7/8 mutants with overexpression of SF3B1 plasmid.

The interaction of SF3B1 with U2AF65 is required for the regulation of *SMN2* pre-mRNA splicing. In addition, better interaction of U2AF65 with the PPT facilitates splicing [5,8]. We have previously demonstrated that the PPT of exon 7 (called PPT7) shows stronger interaction with U2AF65 than the PPT of exon 8 (called PPT8) [18] because PPT7 contains richer pyrimidine nucleotides than PPT8 (Figure 6A). We applied a web-based tool (SVM-BP finder (http://regulatorygenomics.upf.edu/Software/SVM_BP/) [54]) and found that that the PPT7 score (41) was much higher than the PPT8 score (23). Thus, we wondered whether PPT sequences might affect SF3B1 functions on *SMN* exon 7 splicing (Figure 6A). To address this point, we first mutated PPT7 to a weaker one by substituting some uridines in PPT7 with cytidines (called W-PPT7 and had a score of 30) (Figure 6B, left). As shown in Figure 6B (left), SF3B1 could not support cassette exon inclusion in this mutant (lane 3), indicating that weaker PPT hindered SF3B1 function on its exon 7 splicing. We next generated another weaker PPT7 minigene by substituting PPT7 with PPT8 sequences (called E-PPT7/8) (Figure 6B, right). Similar to the W-PPT7 mutant, SF3B1 function on cassette exon splicing was also abolished in this mutant (lane 6). Therefore, weaker PPT could not support SF3B1 function in *SMN2* splicing, indicating that PPT sequences are important for SF3B1 function. These results suggested that weaker U2AF65 binding to PPT7 can interfere with SF3B1 function in *SMN* exon 7 splicing, corroborating that the interaction of U2AF65 with SF3B1 is necessary for SF3B1 function.

### 3.5. SF3B1 Binds to PPT7 More Strongly than PPT8

**Figure 7:**
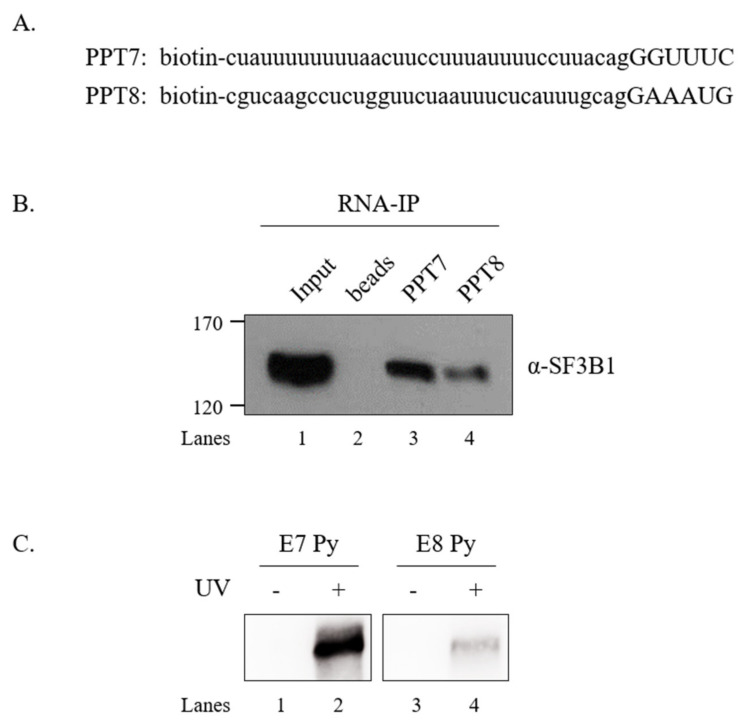
SF3B1 binds to PPT7 more strongly than to PPT8. (**A**) Sequences of biotin-labeled PPT7 and PPT8 oligonucleotides are shown. (**B**) RNA-IP with the anti-SF3B1 antibody using PPT7 and PPT8 oligonucleotides in HEK293T cell lysates. (**C**) UV crosslinking/IP with the anti-SF3B1 antibody using PPT7 and PPT8 oligonucleotides in HEK293T cell lysates.

SF3B1 can interact with upstream and downstream sequences of adenosine (A) nucleotides in BPS, but not with A nucleotides in pre-mRNA [6]. As shown in Figure 5 and Figure 6, the U2AF65 interaction domain of SF3B1 and PPT sequences were important for the regulatory function of SF3B1 in *SMN* exon 7 splicing. We have previously shown that U2AF65 can interact with PPT7 more strongly than with PPT8 [18]. We further wondered whether binding affinities of SF3B1 to PPT7 or PPT8 were different from each other. To this aim, we applied biotin-labeled RNA oligonucleotides of PPT7 and PPT8 that were previously used to analyze the binding of U2AF65 [44] to determine the binding of SF3B1 with two approaches. First, we carried out an RNA-immunoprecipitation (RNA-IP) assay using streptavidin beads, and then performed immunoblotting using the anti-SF3B1 antibody and HEK293T cell lysates. As shown in Figure 7B, SF3B1 could pull down more PPT7 RNAs than PPT8 RNAs (lanes 3 and 4), indicating that SF3B1 could bind to PPT7 more strongly than PPT8 to promote exon 7 splicing or inclusion.

Second, to validate the direct interaction between SF3B1 and PPT, we conducted a UV crosslinking treatment and then performed immunoprecipitation with the anti-SF3B1 antibody followed by immunoblotting with HRP-conjugated streptavidin. As shown in Figure 7C, SF3B1 interacted with PPT7 significantly more than with PPT8 (lanes 2 and 4). These two experiments described above revealed that the PPT sequence with stronger U2AF65 binding also provided more affinity to SF3B1.

## 4. Discussions

As a component of U2 snRNP, SF3B1 has been demonstrated to function in 3′SS recognition through stabilizing the interaction between U2 snRNA and BPS [6,13]. The general role of SF3B1 in pre-mRNA was demonstrated in single-intron splicing. Studies of SF3B1 in AS have focused on disease-causing mutant forms in CLL and MDS, providing gain-of-function evidences of mutant isoforms in alternative usage of BPS and cryptic 3′SS or loss-of-function evidences such as reduced interaction with SUGP1 [21,29,30,31]. However, roles of wild-type SF3B1 in AS are relatively less understood than those of its mutant forms. Here, we studied the roles of wild-type SF3B1 in AS using RNA-seq and SF3B1 KD cells. Our data demonstrated that numerous events of AS, including SEs, A5SS, A3SS, MXE, and RI, were significantly affected by SF3B1, suggesting a widespread function of SF3B1 in AS. Global regulations of SEs, A3SS, and RI by SF3B1 are well-predictable because SF3B1 has important function in 3′SS recognition of constitutive splicing. Both 5′SS and 3′SS recognitions by spliceosomes are affected by each other [46,47,48]. Thus, although SF3B1 is not able to directly regulate 5′SS, 3′SS recognition by SF3B1 might be able to indirectly affect 5′SS.

GO analysis of AS events affected by SF3B1 indicated that various biological functions might be related to SF3B1. We noticed that the function of DNA repair was enriched in SE, A3SS, MXE, and RI categories, suggesting a possible role of SF3B1 in DNA repair. We also found that cancer-related functions such as cell division, regulation of cell cycle, DNA replication, and response to UV were included in altered AS by SF3B1, indicating that wild-type SF3B1 might also play important roles in cancer. In addition, we observed that mitotic nuclear division was enriched in SE, A5SS, and MXE. These functions identified in GO analysis need to be further verified using various experimental approaches. The rMATS tool can be used to provide quantitative alterations of SE, A5SS, A3SS, MXE, and RI between two group of RNA-seq results [51]. Our validation experiments of rMATS analysis showed high validation for the AS events of each category, suggesting a high accuracy of this bioinformatical tool. Among the validated AS events, we observed that the AS of *Fas*, with anti-apoptotic function of cassette exon mRNA and a pro-apoptotic function of exon-included isoform, was reversed by SF3B1 KD. Whether SF3B1 has apoptotic regulatory functions remains to be determined.

Multiple *cis*-elements and transacting factors have been implicated in the regulation of *SMN2* exon 7 splicing [55]. We have demonstrated that SF3B1 can regulate the AS of both *SMN1* and *SMN2* pre-mRNA splicing related to SMA, an autosomal recessive genetic disease [33]. Thus, SF3B1 not only has roles in cancer, but also has roles in genetic diseases. There was a generality of our results in that SF3B1 KD could increase cassette exon skipping with similar effects in HCT116, SH-SY5Y, HEK293T, and even SMA patient cells. Overexpression effects of SF3B1 were also observed in HEK293T and even SMA patient cells. These results revealed that cassette exon splicing could be affected for both *SMN1* and *SMN2* pre-mRNA, suggesting that mutations in *SMN2*, such as C6U transition, are not linked to the role of SF3B1 [39,40]. Therefore, SF3B1 functions differently from the proteins that target the mutation in *SMN2* pre-mRNA, such as serine/arginine-rich splicing factor 1 (SRSF1) and heterogeneous nuclear ribonucleoprotein A1 (hnRNP A1) [56,57]. Similar to SF3B1 protein, hnRNP M, SRC associated in mitosis of 68 kDa (SAM68), serine/arginine-rich splicing factor 2 (SRSF2) and U2AF65 can also regulate *SMN* exon 7 splicing without targeting the mutations in *SMN2* [18,58,59]. Therapeutic approaches of SMA by delivering these genes to cells or knocking down these genes in cells should be considered.

We have previously demonstrated that U2AF65, a PPT-binding protein in 3′SS, can regulate cassette exon splicing of *SMN* through inhibitory activity for intron splicing [44]. In addition, SRSF2 targets the 3′SS of exon 7 to stimulate cryptic 3′SS [59]. Here, we showed that SF3B1, another 3′SS recognition protein, could also function as a regulator of *SMN* exon 7 splicing. These results revealed that 3′SS recognition is a key step in *SMN* exon 7 splicing regulation. It has been shown that BPS, 3′SS, and PPT can bind to proteins or RNA–protein complexes cooperatively to facilitate the recruitment of U2 snRNP to the 3′SS [2]. Interestingly, we found that the U2AF65-binding domain in SF3B1 is required for *SMN2* splicing. This supports that cooperative interactions of proteins with BPS, 3′SS, and PPT are also required for AS. In addition, we demonstrated that a mutation of the PPT at cassette exon to a weaker PPT, which interacts with less U2AF65, could not support SF3B1 function, further indicating the dependency of SF3B1 function on U2AF65. It was recently found that roles of SF3B1 are dependent on SUGP1 [31]. One possibility of the SF3B1 KD effects could be due to loss of SUGP1 interactions needed for proper identification of the BPS.

While SF3B1 can interact with U2AF65 and p14 to support BPS–U2 snRNA interaction, the RS domain of U2AF65 can directly interact with BPS to strengthen base-pairing [4,13]. Although both U2AF65 and SF3B1 can recognize 3′SS, they play opposite roles in *SMN* exon 7 splicing, with U2AF65 showing an inhibitory function and SF3B1 having a stimulatory function in cassette exon inclusion. Therefore, in addition to BPS recognition roles, other unknown functions of SF3B1 and U2AF65 might be involved in regulatory roles of *SMN*.

## Data Availability

The RNA-seq data created in this study is openly available in the NCBI Sequence Read Archive at BioProject accession number PRJNA674660.

## Supplementary Materials

The following are available online at https://www.mdpi.com/2073-4409/9/12/2647/s1, Figure S1: RT-qPCR analysis of *SMN* transcript in SF3B1 KD, non-silencing treated, and untreated HCT116, SH-SY5Y, HEK293T, and GM03813 cells using primers E1F and E1/2aR shown in Figure 4B. Table S1: List of primers used for RT-PCR and RT-qPCR. Table S2: Lists of altered SEs, A5SS, A3SS, and RI by SF3B1 KD.
