# Supplementary figures and images for: U2AF65-Dependent SF3B1 Function in SMN Alternative Splicing
Source: Cells. 2020 Dec 9;9(12):2647. doi: 10.3390/cells9122647 (PMC7762998; doi:10.3390/cells9122647)

Supplementary Figure 1.

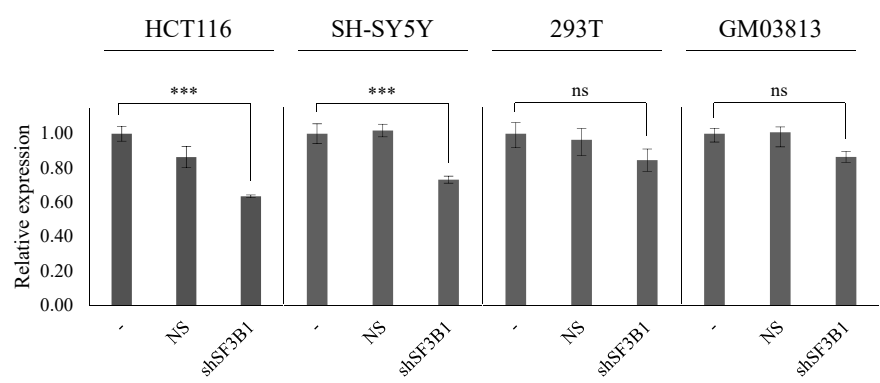

Supplement: Supplementary file 1 [file cells-09-02647-s001.zip › Supplementary Figure S1.pdf]
